# Insulin-like growth factor-II: new roles for an old actor

**DOI:** 10.3389/fendo.2012.00118

**Published:** 2012-10-02

**Authors:** Stefano Cianfarani

**Affiliations:** ^1^Department of Systems Medicine, Tor Vergata UniversityRome, Italy; ^2^Molecular Endocrinology Unit, Bambino Gesù Children’s HospitalRome, Italy

**Keywords:** insulin-like growth factor II, insulin, insulin-like growth factor I, insulin receptor, body composition

## Abstract

Insulin-like growth factor-II (IGF-II), traditionally considered as a growth factor implicated in growth of fetal tissues and cancer cells, is now emerging as a potential metabolic regulator. The aim of this overview is to provide the available evidence, obtained in both experimental conditions and in humans, for a role of IGF-II in the fine-tuning of metabolism and body composition. The underlying mechanisms and the potential clinical implications are discussed.

## IGF-II: A NEGLECTED NODE OF A COMPLEX NETWORK

Insulin-like growth factor-II (IGF-II) is a member of the IGF family of growth factors and related molecules. The IGF family is comprised of three ligands (IGF-I, IGF-II, and insulin), at least six binding proteins (IGFBP-1 to -6), and three specific cell surface receptors that mediate the actions of the ligands [IGF-I receptor, insulin receptor (IR), and the IGF-II mannose-6-phosphate (M-6-P) receptor; Jones and Clemmons, 1995; Cianfarani and Rossi, 1997; Le Roith et al., 2001]. Gene knockout studies revealed that the IGF-I receptor mediates the mitogenic and metabolic actions of both IGF-I and IGF-II, whereas the IGF-II/M-6-P receptor is not considered to have any major role in IGF signal transduction, but is primarily responsible for clearing, and thereby reducing, the levels of IGF-II during fetal development (Baker et al., 1993).

IGF-II is a peptide of 67 amino acids with approximately 50% amino acid homology to insulin (Daughaday and Rotwein, 1989). The human IGF-II gene is located on the short arm of chromosome 11 (11p15.5; Tricoli et al., 1984). This gene is an imprinted gene with paternal allele expressed and maternal allele silenced. Genetic or epigenetic alterations of IGF-II gene are implicated in the pathophysiology of both Beckwith–Wiedemann syndrome and Russell–Silver syndrome (Smith et al., 2007), clearly indicating a key role of IGF-II in prenatal growth. The IGF-II gene comprises nine exons (codons 7–9 being coding) and four promoters, spanning a region of approximately 30 kb (O’Dell and Day, 1998). Several different RNA molecules are formed upon transcription of the gene within the coding region plus one of the various 59-untranslated regions arising from exons 1–6. The different transcripts are expressed according to their tissue and the stage of development.

## “CLASSICAL” PROPERTIES

Albeit *IGF2* expression and IGF-II effects have been reported in several animal and cellular models, the physiological role of this peptide in growth and development still remains largely unknown. IGF-II exerts endocrine, paracrine, and autocrine actions in virtually all tissues (Efstratiadis, 1998). The heterozygous mice carrying a paternally derived mutated IGF-II gene [Igf2(+/p) mutants] and the Igf2(-/-) nullizygotes are phenotypically indistinguishable; they are viable dwarfs with ~60% normal birthweight and, except for a slight delay in ossification, they do not exhibit developmental abnormalities. In contrast, when the disrupted *IGF2* allele is transmitted maternally, the offspring are phenotypically normal, since the maternal allele is normally silent due to imprinting (DeChiara et al., 1990). The overexpression of *IGF2 *increases body size at birth up to 160% (Sun et al., 1997), and size at E17 up to 200% (Eggenschwiler et al., 1997), in a dose dependent manner. Also individual organs can be enlarged in proportion to their IGF-II levels, thus suggesting an autocrine or paracrine control (Sun et al., 1997).

IGF-II exerts a growth promoting action in the placenta. Data originated from mice with placental-specific deletion of P0 promoter of *IGF2*, showed that placental-specific *IGF2 *is required for the attainment of normal placental size and of normal surface area and thickness of the labyrinthine layer where solute exchange takes place in the mouse (Sibley et al., 2004).

Both IGF-I and IGF-II are potent neuronal mitogen and survival factors (Sara et al., 1983; Lund et al., 1986; Adamo et al., 1989). The IGF-I receptor is ubiquitously expressed in all neural cells. The widespread and developmentally associated expression of each component of the IGF system, argues that IGFs act during brain development locally near its sites of expression in an autocrine and/or paracrine fashion. Mutations or deletions of *IGF1* are associated with microcephaly, sensorineural deafness, and mental retardation (Woods et al., 1996; Bonapace et al., 2003; Netchine et al., 2009; Fuqua et al., 2012), a clinical picture consistent with the phenotype of IGF-I gene knockout mice characterized by small brains, hypomyelination, and loss of certain subtypes of neurons (Powell-Braxton et al., 1993). Although IGF-II has been shown to regulate neuronal growth and differentiation in animal models and cell lines (Sullivan et al., 2008; Bracko et al., 2012), only a small proportion of patients with Russell–Silver syndrome with loss of methylation (LOM) of the 11p15 ICR1 telomeric domain (including *IGF2*) leading to reduced IGF-II gene expression in tissues, show developmental delay (Netchine et al., 2007). Interestingly, no single case of mutations/deletions of *IGF2* has been reported so far in humans.

## EVIDENCE FOR UNEXPECTED ACTIONS

Besides the still uncertain physiological roles played by IGF-II in growth and development, there is emerging evidence for new and unsuspected metabolic actions. In humans, *IGF2* has closely been related to the metabolic risk. Several reports have shown that specific polymorphisms of IGF2 are associated with weight and the obese phenotype (O’Dell et al., 1997; Gaunt et al., 2001; Le Stunff et al., 2001; Gu et al., 2002; Zhang et al., 2006). More recently, polymorphisms of IGF-II gene have been related to other cardiovascular risk factors such as fat mass distribution (Rice et al., 2002) and hypertension (Rodríguez et al., 2004; Faienza et al., 2010). In rats, specific *IGF2 *polymorphisms have been associated with hypertriglyceridemia (Kadlecová et al., 2008). These findings are consistent with the mapping of *IGF2* in close proximity to the insulin and tyrosine hydroxylase genes on chromosome 11p15, a genomic region that has been implicated in various common disorders including the metabolic syndrome, type 2 diabetes, and coronary heart disease.

*IGF2* may also play a role in intrauterine programming predisposing to cardiovascular risk in postnatal life. The involvement of IGF-II in programming is suggested by the study of the population exposed to the Dutch Hunger Winter, the period of famine induced by the German-imposed food embargo in the western part of The Netherlands toward the end of World War II in the winter of 1944–1945. The offspring of this population exposed to famine during fetal life showed, decades later, a higher incidence of cardiovascular disease (Painter et al., 2006). Interestingly, a cohort of these individuals prenatally exposed to the Dutch Hunger Winter tested six decades later, showed that the periconceptional exposure to famine was associated with reduced DNA methylation of the imprinted *IGF2 *(Heijmans et al., 2008). This finding suggests that early detrimental cues in critical time windows of development may induce permanent epigenetic changes in *IGF2* probably secondary to a deficiency in methyl donors such as the amino acid methionine. Whether or not these changes in *IGF2 *are associated with an altered expression in the different tissues and are related to the cardiovascular risk has to be established.

In humans, the degree of *IGF2* methylation at birth has recently been related to the development of overweight or obesity in early childhood (Perkins et al., 2012). Interestingly, breastfeeding modified the magnitude of methylation differences between overweight or obese children and children whose weight was within reference range, thus suggesting an interplay between prenatal environment (nutrient transfer from mother to fetus) and early postnatal feeding behavior which could stabilize or change the epigenetic patterns acquired *in utero* (Perkins et al., 2012).

A potential clinical implication of these findings is that *IGF2* methylation may represent an easily assessable marker of intrauterine programming and long-term metabolic risk, thus driving the deprogramming strategies aimed at reducing the metabolic risk in subjects exposed to a suboptimal intrauterine environment.

IGF-II may also act as a metabolic regulator in the interplay between mother and fetus. Specific polymorphisms in paternally transmitted fetal IGF-II gene have recently been associated with increased maternal glucose concentrations in the third trimester of pregnancy and could alter the risk of gestational diabetes in the mother (Petry et al., 2011). These findings are consistent with the Haig’s kinship, or conflict hypothesis (Haig, 1993), according to which the paternally expressed fetal imprinted genes will tend to increase fetal growth, whereas maternally expressed genes will tend to restrain it. This is thought to be achieved by modifying fetal and placental nutritional demand and supply (Reik et al., 2003), including altering maternal glucose concentrations to favor the transfer of glucose from mother into fetus (Petry et al., 2007).

## AN OBSCURE MECHANISM

The mechanisms underlying the observed link between *IGF2* expression and metabolism are largely unknown. As IGF-I and IGF-II share considerable structural homology with insulin (Blundell et al., 1978), it is not surprising that the three peptides can also share biological actions, likely via the IR (King et al., 1980). Indeed, both IGF-I and IGF-II have been shown to stimulate glucose uptake and exert antilipolytic activity in cell cultures (Zapf et al., 1978). The infusion of IGF-II in fasted lambs increases glucose clearance by 15%, whereas no effect was observed on net protein loss or protein synthesis (Douglas et al., 1991).

The human IR exists in two isoforms, isoform A (IR-A) and isoform B (IR-B). Alternative splicing of a small exon (exon 11) of the IR gene results in two slightly different transcripts (Moller et al., 1989). The relative expression of the two isoforms varies in a tissue-specific manner. IR-A is expressed predominantly in central nervous system and hematopoietic cells, while IR-B is expressed predominantly in adipose tissue, liver, and muscle, the major target tissues for the metabolic effects of insulin (Mosthaf et al., 1990). However, IR-A is coexpressed with IR-B in many tissues, especially in muscle and fat (Belfiore et al., 2009). In general, the affinity of IGF-II for IR is low (1–5% that of insulin). However, IGF-II is able to bind with high affinity (30–40% that of insulin) to IR-A. IR-A, when activated by IGF-II, seems to elicit predominantly mitogenic rather than metabolic effects (Frasca et al., 1999; Morcavallo et al., 2011). However, microarray analysis revealed that the majority of genes are regulated similarly by insulin and IGF-II (Pandini et al., 2003). On the basis of these findings, it is tempting to speculate that IR-A mediates the metabolic actions of IGF-II in tissues like muscle and fat.

In pigs, *IGF2* mutations induce changes in body composition characterized by increased muscle mass and reduced backfat thickness (Gardan et al., 2008). In particular, the subcutaneous adipose tissue of animals carrying *IGF2* mutation showed lower lipid content and smaller adipocytes (Gardan et al., 2008). It is noteworthy that adipocytes from subcutaneous and visceral adipose tissues express both IGF-II and IGF-II receptors (Sinha et al., 1990), thus suggesting a physiological role of IGF-II in fat. Consistent with this, IGF-II stimulates preadipocyte proliferation *in vitro* (Siddals et al., 2002). In mutant mice, the loss of both *IGF2* and Myod genes induces massive brown adipose tissue hypertrophy compared with wild-type and single-mutant newborns. The concomitant Myod and *IGF2* inactivation accelerates differentiation of a brown preadipocyte cell line and induces lipid accumulation (Borensztein et al., 2012).

Furthermore, IGF-II may regulate body composition and affect metabolic risk factors by controlling muscle mass. IGF-II has also been shown to stimulate skeletal myoblast differentiation and myofiber hypertrophy (Florini et al., 1991; Stewart and Rotwein, 1996; Stewart et al., 1996) thus concurring to skeletal muscle growth and development (Van Laere et al., 2003; Alzhanov et al., 2010).

**FIGURE 1:**
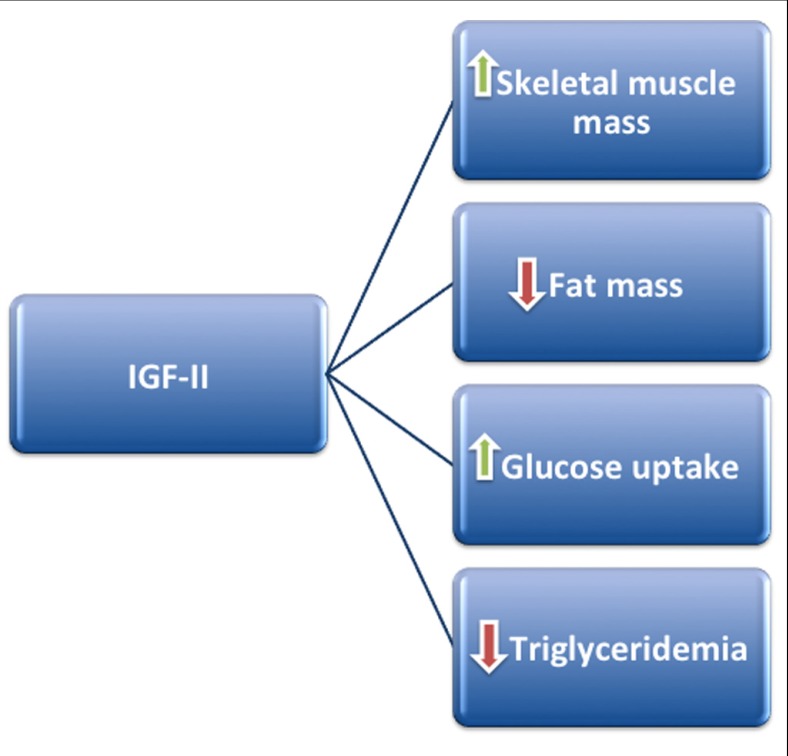
**IGF-II may positively regulate metabolism by stimulating skeletal muscle mass accrual and glucose uptake, at the same time decreasing fat mass and triglyceride production**.

These experimental data, obtained in animal and cellular models, argue that IGF-II may regulate metabolic homeostasis by affecting body composition, favoring skeletal muscle accrual probably at expense of adipose tissue, and ultimately leading to a phenotype less prone to cardiovascular risk (**Figure 1**).

## CONCLUDING REMARKS

IGF-II was characterized almost four decades ago but its physiological role is still largely unknown. The data reported above strongly suggest a metabolic role of this growth factor. Although the evidence is still weak, the efforts to elucidate the IGF-II metabolic actions in tissues, especially in muscle and fat, appear worthwhile as they could open avenues for understanding the interplay between IGF-II and insulin in both physiological and pathological conditions. Finally, data in humans suggest that *IGF2* polymorphisms or epigenetic changes may represent a marker of metabolic risk to be exploited for selecting the individuals to be targeted with specific nutritional and/or pharmacological prevention strategies.

## Conflict of Interest Statement

The author declares that the research was conducted in the absence of any commercial or financial relationships that could be construed as a potential conflict of interest.
